# Optimization of treatment with interferon beta in multiple sclerosis. Usefulness of automatic system application criteria

**DOI:** 10.1186/1471-2377-8-3

**Published:** 2008-03-06

**Authors:** Juan Luís Ruiz-Peña, Pablo Duque, Guillermo Izquierdo

**Affiliations:** 1 Unidad de Esclerosis Múltiple, Hospital Universitario Virgen Macarena, Avda, Dr. Fedriani 3, 41009 – Sevilla, Spain

## Abstract

**Background:**

A software based tool has been developed (Optem) to allow automatize the recommendations of the Canadian Multiple Sclerosis Working Group for optimizing MS treatment in order to avoid subjective interpretation.

**Methods:**

Treatment Optimization Recommendations (TORs) were applied to our database of patients treated with IFN β1a IM. Patient data were assessed during year 1 for disease activity, and patients were assigned to 2 groups according to TOR: "change treatment" (CH) and "no change treatment" (NCH). These assessments were then compared to observed clinical outcomes for disease activity over the following years.

**Results:**

We have data on 55 patients. The "change treatment" status was assigned to 22 patients, and "no change treatment" to 33 patients. The estimated sensitivity and specificity according to last visit status were 73.9% and 84.4%. During the following years, the Relapse Rate was always higher in the "change treatment" group than in the "no change treatment" group (5 y; CH: 0.7, NCH: 0.07; p < 0.001, 12 m – last visit; CH: 0.536, NCH: 0.34). We obtained the same results with the EDSS (4 y; CH: 3.53, NCH: 2.55, annual progression rate in 12 m – last visit; CH: 0.29, NCH: 0.13).

**Conclusion:**

Applying TOR at the first year of therapy allowed accurate prediction of continued disease activity in relapses and disability progression.

## Background

Disease Modifying Treatments (DMTs) give neurologists a tool for treating multiple sclerosis (MS). There is evidence that these DMTs reduce disease activity and change the natural course of MS, but in clinical practice it is difficult to control the magnitude of the treatment effect and to evaluate treatment responsiveness in concrete cases. Currently, clinicians do not have criteria for defining suboptimal responses.

The Canadian Multiple Sclerosis Working Group (CMSWG) developed practical recommendations [1] on how neurologists should assess the status of patients on DMT and help them to decide when optimizing treatment might be necessary. Nevertheless, these recommendations need prospective validation.

We have developed a software based tool to allow us score the recommendations of the CMSWG, avoiding a subjective interpretation of the results, using disease status assessments. Our objectives are (a) to validate the Treatment Optimization Recommendations (TORs) established by the CMSWG, using an automatic program developed by our group in application to a Spanish population of definite relapsing-remitting MS (RRMS) patients initially treated with interferon beta 1a, 30 mg/week IM; and (b) to evaluate whether TOR disease status assessments (based on relapses and disability progression) after one year of treatment with low dose interferon beta 1a can predict clinical outcomes after years of treatment.

## Methods

### Study population

We reviewed 55 patients from the database of the MS Clinic of Hospital Universitario Virgen Macarena (Seville, Spain) who were diagnosed of RRMS according to the criteria of Poser et al [2], and treated with Interferon β 1a IM 30 mg/week. These patients started treatment between January 1998 and May 2004, and were subjected to prospective follow-up. Patients were evaluated at least four times a year to assess disability, and the number and severity of relapses. Disability was measured by trained neurologists using Kurtzke's EDSS [3]. All patients gave written informed consent for storage of their data in a central database for research purposes and the study was approved by the hospital's ethical committee.

### Optem

Optem is a software tool developed by our group, allowing easy and objective automatic optimization of treatment changes based on the CMSWG criteria. The program comprises three screens – one for each field examined: attacks, progression and magnetic resonance imaging. On completing these fields, the program automatically develops a risk report based on the information supplied in each screen. Following the optimization criteria of the CMSWG, and concluding whether the patient must be optimized towards other treatment or can continue with the current therapy.

In the screen "attacks" the application assesses 3 parameters: relapse reduction, relapse recovery and severity, scoring from 0 to 3 points according to null, low, medium or high level.

In order to determine the relapse reduction, the software calculates the "annual relapse rate" for each period. The number of relapses is registered for each period and is considered to be "Low" for a relapse rate reduction between 75 and 99%, "medium" for a relapse rate reduction between 35 to 74% and "high" if the result ranges between a 0 and 34%.

Relapse recovery is considered to be "low" if there is a prompt recovery, "medium" if recovery is incomplete at 3 months and "high" if recovery is not complete at 6 months.

Relapse severity is considered to be "mild", "moderate" or "serious" depending on corticosteroids use, hospitalization, functional systems affected and activities of daily living.

In the screen "progression", disability progression is assessed under two categories: EDSS and clinical progression. It is considered low, medium or high with according to EDSS progression and the presence of sensitive, motor, cerebellar or cognitive symptoms.

The screen "MRI" evaluates changes in imaging parameters and classifies as "none" for no change, "low" if there are changes in 2 categories, "medium" for changes in 3 categories and "high" for changes in more than 3 categories. The six categories evaluated are: Gadolinium enhancing lesions, new T2 lesions, T2 burden of disease, new T1 lesions, T1 burden of disease and brain atrophy.

This information is stored and can be shown on-screen to graphically illustrate the evolution of the risk reports on a given patient and his or her tendency to maintain or worsen disease status.

### Clinical assessments

TORs were applied retrospectively to our patients. The criteria used were based on the CMSWG modified analog model, but imaging data were not considered because MRI was not performed in all cases, following Freedman et al recommendation when MRI is no available [4]. Patient data were assessed during year 1 for disease activity by relapse and progression rate, and subjects were assigned to two groups according to TOR. The levels of concern were "change treatment" (CH) and "no change treatment" (NCH). According to the analog model, if all three items were "low", two of them were "medium" or any one of them was "high", then it was likely that treatment response might be suboptimal and patients were therefore assigned to the "change treatment" group.

These assessments were then compared to observed clinical outcomes for disease activity over the following years.

Patients were evaluated until the neurologist decided to change or discontinue the immunomodulatory treatment.

To evaluate the patient evolution in each group, the number of relapses per year and EDSS were calculated for each year (2^nd^, 3^rd^, 4^th ^and 5^th^) and patient, and the means for each group (CH or NCH) were compared. We also used the relapse rate and the progression rate for the interval of 4 years (2^nd ^to 5^th^), to compare both groups.

### Statistical analysis

Data were processed using the SPSS version 11.0.0 statistical package, and a descriptive and analytical statistical analysis was carried out.

The mean, standard deviation (SD), median, and the upper and lower quartiles were used for the description of numerical variables. A comparison between the "change treatment" (CH) group and the "no change treatment" (NCH) group was made in relation to relapse and progression data, using univariate analysis of variance (ANOVA).

The sensitivity of the algorithm was calculated as the number of patients considered as CH (need for change) by the tool amongst the total population of patients considered as non-responders after the evolution period.

The specificity of the algorithm was calculated as the number of patients who responded to treatment in relation to the patients considered as NCH ("no need for change").

## Results

### Subject demographics and clinical parameters

We have data on 55 patients (10 males, 45 females) assigned to therapy with IFN beta 1a IM for 51 months (range 18 – 84). Data from other DMTs were excluded due to the required homogeneity in therapy in a validation study of treatment response criteria. The mean number of relapses within the previous two years was 2.5 (range 2–7), and the baseline EDSS was 1.8. "Change treatment" (CH) status was assigned to 22/55 patients (40%), and "no change treatment" (NCH) to 33 patients (60%). Table 1 displays the demographic and clinical characteristics of the two patient populations, showing no differences in baseline characteristics.

**Table 1:** Clinical data of patients before treatment in both groups (p > 0.05)

	**CHANGE (CH)**	**No Change(NCH)**
Number	22	33
Time to treatment (months)	52.5	51.2
EDSS before treatment	1.86	1.87
Attacks 2 yr. before treatmen	2,7	2.3
Attacks 1 yr. before treatmen	1.9	1.9
Age at onset	27.3	27.0
Duration of disease	13.7	12.6

The number of patients analysed in each period were 55 in the 1^st ^and 2^nd ^year, 44 in the 3^rd ^year, 34 in the 4^th^, and 23 in the 5^th ^year. During the first two years of therapy (one year before and one year after optimization), all patients continued with the same treatment, dose and route of administration. During the third year, 10 patients switched treatment, versus 12 and 9 patients in the fourth and fifth years, respectively. At the end of the follow-up period, 9 out of 22 (41%) patients of the NCH group and 14 of 32 (44%) of the CH group continued with the same treatment, dose and route of administration. These differences were not statistically significant. In those cases where treatment was changed to a higher dose, this was not attributable to side effects; but rather, from the application of the treatment criteria of the Andalusian health authorities to the clinical evolution parameters in relation to relapses and progression. Despite this, there were no losses in terms of patient follow-up. All patients initially evaluated continued follow-up at the MS Unit with the same or different therapy.

### Sensitivity and specificity

The estimated sensitivity according to the last visit status of every patient was 73.9%. In turn, specificity was 84.4%, and the positive predictive value was 77.3%.

### Relapse rate during year 2–5

**Figure 1:**
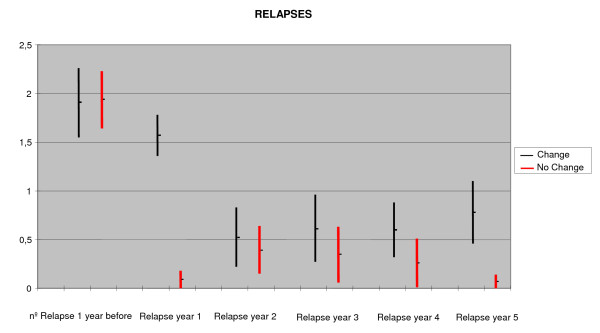
Relapse rate during year 0 – 5 (95% Confidence Interval).

Figure 1 shows the mean number of attacks during the complete period (2^nd ^to 5^th ^year). The differences appeared after the second year (0.52 ± 0.67 vs. 0.39 ± 0.7, p = 0.5, CH vs. NCH, respectively), and increased after the third year (0.61 ± 0.77 vs. 0.34 ± 0.68, p = 0.24 CH vs. NCH, respectively) and fourth year (0.60 ± 0.51 vs. 0.26 ± 0.56, p = 0.07 CH vs. NCH, respectively), but were not statistically significant until the 5^th ^year (0.7 ± 0.66 vs. 0.07 ± 0.26, p = 0.001 CH vs. NCH, respectively).

On considering the global follow-up period of those patients who completed follow-up until the 5^th ^year without changing treatment, and the period covering from years 2 to 5, the differences were seen to be clearly significant (CH: 1.72 ± 1.58, NCH: 0.85 ± 1.20, p = 0.02).

### Progression during year 2–5

**Figure 2:**
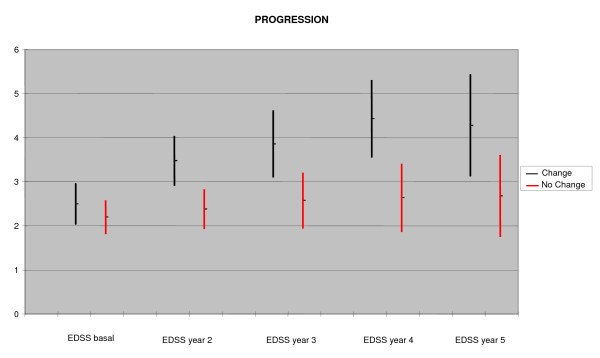
Progression of disability from year 2 to year 5. (95% Confidence Interval, EDSS: Expanded Disability Status Scale).

On analyzing EDSS progression (Figure 2), we observed a statistically significant difference from the second year onwards (3.47 ± 1.45 in the CH group vs. 2.37 ± 1.17 in the NCH group, p = 0.003). This difference was maintained after the third (3.86 ± 1.67 in the CH group vs. 2.57 ± 1.54 in the NCH group, p = 0.01), fourth (4.42 ± 1.69 in the CH group vs. 2.63 ± 1.54 in the NCH group, p = 0.003) and fifth year of follow-up (4.28 ± 1.6 in the CH group vs. 2.68 ± 1.7 in the NCH group, p = 0.04). The annual progression rate (EDSS points/year) from the 12-month visit to the last visit was higher in the group "change treatment" as compared to the "no change treatment" group (CH: 0.29 ± 0.34, NCH: 0.13 p = 0.03).

## Discussion

The lack of criteria for DMT efficacy in MS is an important and unresolved issue. Several DMTs are now available for the treatment of MS, and have been shown to alter the clinical course of the disease by decreasing activity and delaying the progression of disability. Nevertheless, many patients continue to experience disease activity whilst on treatment, and recommendations have been made on how the success of therapy in an individual patient can be assessed. However, even after having identified criteria for a suboptimal response to current treatments, clinicians require guidance on how to improve the outcomes [5]. First Bashir et al. [6] and subsequently a consensus group [1] addressed this problem with objective clinical criteria. The main recommendation of this consensus group is to collect data as soon as possible, in order to allow neurologists to change therapy when treatment is considered to be ineffective. With our program, the Canadian group criteria can be applied prospectively, easily and objectively.

In our study, patients treated with interferon beta 1a IM, 30 mg/week, might potentially be considered as candidates for treatment change according to TOR, after one year of treatment and without including MRI data. The results presented by Freedman et al [4] were different when Rebif 22 was used, and only 10–12% of 362 patients were considered to be candidates for change.

The annualized relapse rate of those patients on Avonex from year 2 to year 5 was 1.72 for those assigned to the "CH treatment group" and 0.84 for these assigned to the "NCH treatment group". In the work of Freedman et al., the combined annualized relapse rate for patients on Rebif 22 or 44 during years 2 to 4 was 0.48 for those with no or low concern during year 1, and 1.12 for those assigned to medium or high concern. Most patients in both subgroups treated with Rebif (48% on 22 mg dose and 53% on 44 mg dose) assigned to the high concern status had more than one attack per year. These results are similar to our own data. The differences in the classification of the status of concern, and the different doses and route of administration of interferon beta 1a may make difficult additional comparisons between the study by Freedman et al. and our own data.

Our data showed that treatment optimization recommendations applied at the end of the first year of therapy based exclusively on clinical outcomes are useful to predict a high level of clinical activity (relapses and disability), with a high sensitivity and specificity.

Surprisingly, in our study the tool showed to be more effective in predicting EDSS progression rather than changes on the relapse rate. At the end of the first year of follow-up (2^nd ^year after the start of treatment), when all 54 patients continued with the same treatment, the difference on EDSS was already seen (3.47 points in the high risk group versus 2.37 points, with a high level of statistical significance, p = 0.003, after the second year). At the end of the third year, the difference was similar, though the level of statistical significance decreased because 10 patients changed treatment and were not considered in the statistical analysis. After the third year, the differences proved to be stable, although the level of statistical significance decreased further still, because of the fewer remaining patients in the sample (Figure 2).

We lost a similar number of patients in both groups, and we are unable to explain this observation. In fact, we expected to have more losses in the CH group because of a poorer disease evolution. Anyhow, it is clear that those patients considered by the algorithm as candidates for change, who continue with the same treatment, are at high risk of a poorer course than those considered as non-candidates for change.

The lack of significant differences on the relapse rate until the fifth year of treatment can be due to the fact that a clear reduction of the relapse rate was observed in both groups.

Freedman et al. [4] found no statistically significant differences on EDSS parameters from years 2 to 4, though it is necessary to point out that 19% of the patients treated with Rebif 22 and only 5% of those treated with Rebif 44 progressed more than 1 point for EDSS. The differences in the dose, route and frequency of administration of IFNbeta 1a of Avonex and Rebif, could partly explain these differences.

We agree with Freedman et al. that TOR is a good tool for monitoring the treatment response to DMT in MS patients. Our program is easy to use and not time-consuming, and therefore can help neurologists to evaluate patients objectively and easily.

We think that additional items can be explored as potential predictors of treatment response, such as cerebrospinal fluid parameters, IgG synthesis [7], IgG or IgM oligoclonal bands [8,9], and anti-interferon beta antibodies, and can also be included in new versions of the software, in addition to MRI parameters.

### Study limitations

One of the limitations of the study might be the fact that is based on a post-hoc analysis. Even though the clinical data (EDSS score and relapse rate) were obtained prospectively, the optimization criteria were recorded in a retrospective way in the software.

Another limitation could be the fact that the sample size was too small to draw strong conclusions.

In this study we have a pre-treatment control period of two years. This period could be considered to be longer than required, since such a long pre-treatment period could underestimate the annualized relapse rate. If we would use a control period of one year, it is likely that fewer patients would be considered as candidates to change (CH), because the pre-treatment relapse rate would be higher. But we do not think that this fact modifies the results from our study. Nevertheless, we consider that in the future, new prospective studies with different pre-treatment control periods (one to three years) should be conducted.

We have shown that TOR, is a good tool for monitoring the response to DMT in MS patients, and can help optimize treatment. Our program would help neurologists in clinical practice, because it is not time-consuming and easy to apply – providing a tool for objective and simple patient assessment. Optem also can be a useful tool for prospective follow-up studies.

## Conclusion

1. Treatment optimization recommendations is a good tool for monitoring the treatment response to DMT in MS patients: Our data showed that TOR applied at the end of the first year of therapy based exclusively on clinical outcomes are useful to predict a high level of clinical activity (relapses and disability), with a high sensitivity and specificity.

2. Our program is easy to use and not time-consuming, and therefore can help neurologists to evaluate patients objectively and easily.

## Competing interests

The author(s) declare that they have no competing interests.

## Authors' contributions

GI conceived the study, and participated in its design and coordination. JLRP drafted the manuscript. PD designed the software. All authors read and approved the final manuscript.

## Pre-publication history

The pre-publication history for this paper can be accessed here:


